# The Morton Fund Association

**Published:** 1864-01

**Authors:** 


*The Morton Fund Association.*—A systematic and determined effort is being made by the physicians of Boston, to obtain an appropriation by Congress, for Dr. Morton, the alleged discoverer of Ætherization, to reimburse him for outlays in discovering, defending his claim—and vainly endeavoring to obtain some pecuniary consideration, from the same source—and to remunerate him for the great benefits derived from the discovery, by the public and especially by our soldiers in the field. The association having the matter in charge comprises many of the leading physicians of Boston. They hope to influence the action of Congress by inducing physicians throughout the country to exercise their personal influence on the Representatives from their particular district. It will be well for Dr. Morton if Congress can be led to act upon the principle of the adage, "*Bis dat qui cito dat.*"

